# Simultaneous copy number gains of *NUPR1* and *ERBB2* predicting poor prognosis in early-stage breast cancer

**DOI:** 10.1186/1471-2407-12-382

**Published:** 2012-08-31

**Authors:** Seung-Hyun Jung, Ahwon Lee, Seon-Hee Yim, Hae-Jin Hu, Chungyoul Choe, Yeun-Jun Chung

**Affiliations:** 1 Integrated Research Center for Genome Polymorphism, College of Medicine, The Catholic University of Korea, 505 Banpo-dong, Socho-gu, Seoul, 137-701, Republic of Korea; 2 Department of Microbiology, The Catholic University of Korea, Seoul, South Korea; 3 Department of Hospital Pathology, Humanities and Social Sciences, The Catholic University of Korea, Seoul, South Korea; 4 Department of Medical Humanities and Social Sciences, The Catholic University of Korea, School of Medicine, 505 Banpo-dong, Seocho-gu, Seoul, 137-701, South Korea

## Abstract

**Background:**

The full extent of chromosomal alterations and their biological implications in early breast carcinogenesis has not been well examined. In this study, we aimed to identify chromosomal alterations associated with poor prognosis in early-stage breast cancers (EBC).

**Methods:**

A total of 145 EBCs (stage I and II) were examined in this study. We analyzed copy number alterations in a discovery set of 48 EBCs using oligoarray-comparative genomic hybridization. In addition, the recurrently altered regions (RARs) associated with poor prognosis were validated using an independent set of 97 EBCs.

**Results:**

A total of 23 RARs were defined in the discovery set. Six were commonly detected in both stage I and II groups (> 50%), suggesting their connection with early breast tumorigenesis. There were gains on 1q21.2-q21.3, 8q24.13, 8q24.13-21, 8q24.3, and 8q24.3 and a loss on 8p23.1-p22. Among the 23 RARs, copy number gains on 16p11.2 (*NUPR1*) and 17q12 (*ERBB2*) showed a significant association with poor survival (*P* = 0.0186 and *P* = 0.0186, respectively). The patients simultaneously positive for both gains had a significantly worse prognosis (*P* = 0.0001). In the independent replication, the patients who were double-positive for *NUPR1-ERBB2* gains also had a significantly poorer prognosis on multivariate analysis (HR = 7.31, 95% CI 2.65-20.15, *P* = 0.0001).

**Conclusions:**

The simultaneous gain of *NUPR1* and *ERBB2* can be a significant predictor of poor prognosis in EBC. Our study will help to elucidate the molecular mechanisms underlying early-stage breast cancer tumorigenesis. This study also highlights the potential for using combinations of copy number alterations as prognosis predictors for EBC.

## Background

Breast cancer is the most common female cancer and the leading cause of cancer-related mortality in women worldwide [1,2]. Due to mammographic screening and advances in chemotherapy, breast cancer mortality rates have decreased in developed countries since 1990 [3,4]. Nonetheless, axillary-node negative patients treated by surgery showed a ten-year recurrence rate of approximately 20% [5]. The five-year survival rate of stage I and II breast cancer patients is reported to be approximately 80% to 88% [6-8]. This means that 10-20% of early-stage breast cancer (EBC) patients have poor clinical outcomes. When considering the large impact that breast cancer has on public health, it is worth investigating genetic mechanisms underlying poor clinical outcomes of some EBCs.

Genomic instability is one of the hallmarks of breast cancer. DNA copy number aberrations, commonly detected phenomena in cancer lesions, are thought to be involved in tumorigenesis and to affect cancer phenotypes [9]. Different patterns of copy number alterations are associated with distinct gene expression patterns and clinical characteristics of breast cancer [10]. A number of chromosomal alterations and subsequent expression changes have been investigated to determine their implications in clinical phenotypes or prognosis. These investigations have resulted in the identification of some cancer-related genes in breast cancer [11]. For example, HER2 amplification/overexpression is known to occur at an early developmental stage of ductal carcinoma in situ (DCIS). Loss of 16q, where potential tumor suppressor genes such as E-cadherin (*CDH1*) and *CDH13* are located, is also known to be a major event in low-grade invasive ductal carcinoma [12,13]. Especially, recent larger-scale studies have elucidated the molecular complexity of breast cancer and suggested novel genetic subgroups [14-18]. However, since most of them studied Caucasians or Hispanics, the profiles of chromosomal alterations and their biological implications in Asians are relatively less well studied.

In this study, we aimed to describe commonly occurring chromosomal alterations in EBC (stage I and II) and to explore the implications of recurrently altered regions (RAR) on patient prognosis. For this purpose, we analyzed DNA copy number alterations (CNAs) across the whole genome using oligoarray-comparative genomic hybridization (CGH) in a discovery set of EBC patients. RARs in the discovery set that were found to be significantly associated with prognosis were validated in an independent replication set. Our results will contribute to a better understanding of early tumorigenesis in breast cancer and will help to predict the prognosis of EBC patients.

## Methods

### Patients and tumor specimens

As a discovery set for the whole genome array-CGH analysis, frozen tumor tissues were obtained from 48 EBC patients who underwent surgical resection at Dankook University Hospital in Cheonan, Korea (from 1998 to 2002). As an independent replication set, 97 formalin-fixed, paraffin-embedded (FFPE) EBC tissue samples (from 1996 to 2002) were obtained from Seoul St. Mary’s Hospital, Korea. Patient survival status was obtained in 2010 from the Korean Central Cancer Registry, Ministry of Health and Welfare, Korea. All breast cancers were stage I, IIA, or IIB. This study was performed under approval from the Institutional Review Board of the Catholic University Medical College of Korea (CUMC06U015). Tumor stage was determined according to the standard AJCC guidelines for tumor-node-metastasis classification (sixth edition). Clinicopathologic characteristics of the study subjects are summarized separately for the discovery and replication sets in Table 1. Hormone receptor status for ER, PR and HER2 was obtained through a medical record review and for the cases without the hormone receptor status, immunohistochemical (IHC) staining for ER, PR and HER2 was performed. Based on the IHC measurement, breast cancer cases were categorized into four different molecular subtypes as described elsewhere: luminal type A (ER + and/or PR +, HER2 -: Luminal A), luminal type B (ER + and/or PR +, HER2 +: Luminal B), Her2 overexpressed (ER - and PR -, HER2 +: HER2), and triple negative (ER -/PR -/HER2 -: TNBC) [19]. For array-CGH analysis, 10-μm-thick frozen sections of tumor cell-rich areas (>60%) were microdissected. Genomic DNA was extracted from these sections using a DNeasy Blood & Tissue Kit (Qiagen, Hilden, Germany). For genomic real-time quantitative PCR (qPCR) analysis, 10-μm-thick paraffin sections of tumor cell-rich areas (>60%) in the replication set were microdissected. After paraffin removal, genomic DNA was extracted using a DNeasy Blood & Tissue Kit (Qiagen). Genomic DNA from a healthy female individual was used as the normal reference for all array-CGH experiments. Genomic DNA extracted from the blood of a Korean female individual without breast cancer was used as universal normal reference for all the array-CGH experiments.

**Table 1:** General characteristics of the study subjects

**Characteristics**	**Discovery set**	**Replication set**
	**(n = 48)**	**(n = 97)**
**Age group**		
< 50 years	26(54.2%)	22(45.8%)
≥ 50 years	48(49.5%)	49(50.5%)
**Stage**		
Stage I	11(22.9%)	25(25.8%)
Stage II	37(77.1%)	72(74.2%)
Stage IIA	26	50
Stage IIB	11	22
**ER status**		
Positive	25(52.1%)	54(55.7%)
Negative	23(47.9%)	43(44.3%)
**PR status**		
Positive	35(72.9%)	59(60.8%)
Negative	13(27.1%)	38(39.2%)
**HER2 status**		
Positive	11(22.9%)	23(23.7%)
Negative	37(77.1%)	74(76.3%)
**Subtype**		
Luminal A	29(60.4%)	53(54.6%)
Luminal B	10(20.8%)	16(16.5%)
HER2	1(2.1%)	7(7.2%)
TNBC	8(16.7%)	21(21.6%)

### Array-CGH and data processing

For array-CGH analysis, 30K whole-genome human oligoarrays (Human OneArray^TM^; Phalanx Biotech, Palo Alto, CA) were used. Oligoarray-CGH was performed as described elsewhere [20]. In brief, 1 μg of genomic DNA from tumor tissue was labeled with Cy3-dCTP. The reference DNA was labeled with Cy5-dCTP (GeneChem, Daejon, Korea). Dye-labeled DNA was purified with BioPrime spin columns (Invitrogen, Carlsbad, CA) and precipitated with 100 μg of human Cot-1 DNA (ConnectaGen, Seoul, Korea). The labeled DNA pellet was dissolved in 50 μl of DIG hybridization buffer (Roche, Mannheim, Germany), to which 600 μg of yeast t-RNA (Invitrogen) was added. The labeled DNA solution was applied on the array and incubated for 48 hours at 37°C in a MAUI hybridization machine (BioMicro, Salt Lake City, UT). After washing the slides, arrays were scanned using a GenePix 4000B scanner (Axon Instruments, Sunnyvale, CA) and feature extraction was performed using GenePix Pro 6.0. Normalization and re-alignment of raw array CGH data were performed using the web-based array CGH analysis interface, ArrayCyGHt [21]. A print-tip Loess normalization method was used and each probe was mapped according to its genomic location in the UCSC genome browser (Human NCBI36/hg18). In total, 24,107 probes were processed out of initial 26,616 probes. Array-CGH data for all 48 cancers are available through GEO (accession no GSE37839).

### Detection of recurrent copy number alterations

The rank-segmentation statistical algorithm in NEXUS software v3.1 (BioDiscovery Inc., El Segundo, CA) was used to define CNAs of each sample. To optimize the algorithmic parameters for calling CNAs, 11 independent normal-to-normal hybridizations were performed (10 self-to-self and 1 male-to-female hybridizations). The parameters for defining CNAs were as follows: significance threshold = 5.0E-4; maximum contiguous probe spacing (Kbp) = 1000; minimum number of contiguous probes per CNA segment = 5; threshold of signal intensity ratio >0.2 on log_2_ scale for gains and < −0.3 on log_2_ scale for losses. After defining CNAs, RAR was determined to be the chromosomal segment covering overlapping CNAs that appeared in at least 30% of the samples with *P* < 0.05 in the discovery set (NEXUS software v3.1). High-level amplification (amplification hereafter) was defined as a probe signal intensity ratio of 1.5 or higher on the log_2_ scale. Likewise, a homozygous deletion (HD) was defined as a ratio of −1.5 or lower on the log_2_ scale.

### Genomic quantitative PCR analysis

qPCR validation of the significant RARs was performed using genomic DNA extracted from the FFPE samples of 97 EBCs. As a diploid internal control, a genomic region on chromosome13 (13q32.1) that showed no genomic alteration in the array-CGH data was used. Details including primer information for targets and the diploid control locus are available in Additional file 1. Genomic qPCR was performed using the Mx3000P qPCR system (Stratagene, La Jolla, CA), as described elsewhere [22]. In brief, a 10-μl real-time qPCR mixture containing 10 ng of genomic DNA, SYBR Premix Ex Taq II^TM^ (TaKaRa Bio, Japan), 1× ROX, and 5 pmole of each primer was prepared. Thermal cycling conditions consisted of one cycle of 30 sec at 95°C followed by 45 cycles of 5 sec at 95°C, 10 sec at 55–61°C, and 20 sec at 72°C. All qPCR experiments were repeated three times and relative quantification was performed by the ΔΔCT method. When mean genomic dosage ratios of the region between the target sample and female control DNA (ΔΔCT of target and internal control) were above two, the region was defined as a copy number gain.

### Association rule mining

The association rule mining is used for finding interesting relations among variables in a database. In bioinformatics, the information metric was commonly used to assess the degree of “surprise” when a pattern actually occurs [23]. We used CPAR (Classification based on Predictive Association Rules) [24] algorithm adopting the information metric which was implemented by the LUCS-KDD research group (http://www.csc.liv.ac.uk/~frans/KDD/Software). In CPAR, *Laplace accuracy* is used to measure the accuracy of rules. Given a rule *r*, *Laplace accuracy* is defined as follows:

(1) $$\text{Laplace}\;\text{accuracy}\left(r\right)=\frac{\left(N_{c}+1\right)}{\left(N_{\text{total}}+m\right)}$$

where *m* is the number of classes and *N*_*total*_ is the total number of examples that satisfy the rule’s body, among which *N*_*c*_ examples belong to the predicted class, C of the rule.

Through the CPAR algorithm, association rules were generated between RAR markers and the survival status in the discovery set. Each RAR marker was coded as 0 or 1 based on the copy number status; 0 indicates no copy number variation and 1 indicates copy number change in the marker region. Likewise, the survival status was coded as 0 (dead) or 1 (alive).

### Statistical analysis

To examine the clinicopathologic implications of RARs, five clinical parameters were used as categorical variables: age at diagnosis (<50 *vs.* ≥50 years), stage, ER status, PR status, and HER2 status. Differential distributions of RARs in each category were tested by a two-sided Fisher’s exact test. The false discovery rate (FDR) was used for multiple comparison correction. In univariate survival analysis, cumulative overall survival was calculated according to the Kaplan-Meier method. Differences in survival curves were assessed with the log-rank test. Cox regression was performed to identify RARs associated with prognosis after adjusting for age, stage, ER, PR, and HER2. SAS version 9.1 (SAS Institute Inc., NC) was used and *P*-values less than 0.05 were considered significant in all statistical analyses.

## Results

### General characteristics of copy number alterations in EBC

**Figure 1:**
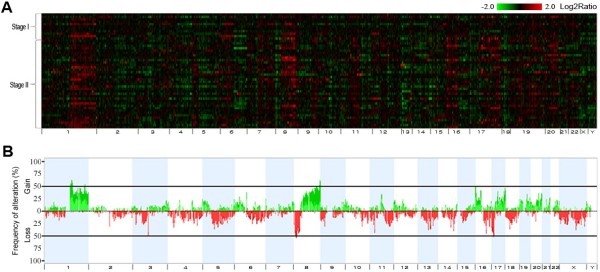
**Genome-wide profiles and frequency plot of chromosomal alterations in 48 breast cancer samples.** (**A**) The genomic alteration profiles of 48 early breast cancers are presented in individual lanes. A total of 24,107 probes are mapped according to the UCSC genome browser (Human NCBI36/hg18) and ordered by chromosomal position from 1pter to Yqter (X-axis). Tumor *vs.* reference intensity ratios are plotted in different color scales reflecting the extent of genomic gains (red) and losses (green), as indicated on the reference color bar (log_2_ scale). (**B**) Frequencies of copy number gains and losses in the 48 breast cancers. The green bars denote the copy number gains and the red bars denote the copy number losses. Boundaries of individual chromosomes are indicated by vertical bars.

Genome-wide CNAs of the 48 EBCs (discovery set) were examined individually, as described in the Methods (Figure 1A). The median number of CNAs per each sample was 10 (range 1–21). Frequency plots of CNAs in the discovery set showed that alterations were not randomly distributed, but were clustered in several hot regions across the whole chromosomes (Figures 1A and B). The overall CNA frequency profiles were similar for stage I and II groups, and among the molecular subtypes (Additional file 1: Figure S1). Of all 4,396 CNAs, copy number gains on 16p13.3, 16p12.3, and 17q25, and copy number losses on 16q21, 17p12, 17p13, and 20q11.1 were significantly more frequent in the stage II groups based on unadjusted *P* values (*P* < 0.05), but none after multiple comparison correction (Additional file 1: Table S2). On average, 376 Mb (13% of the whole genome) per EBC tissue specimen showed chromosomal alterations.

### Recurrently altered chromosomal regions in EBC

**Figure 2:**
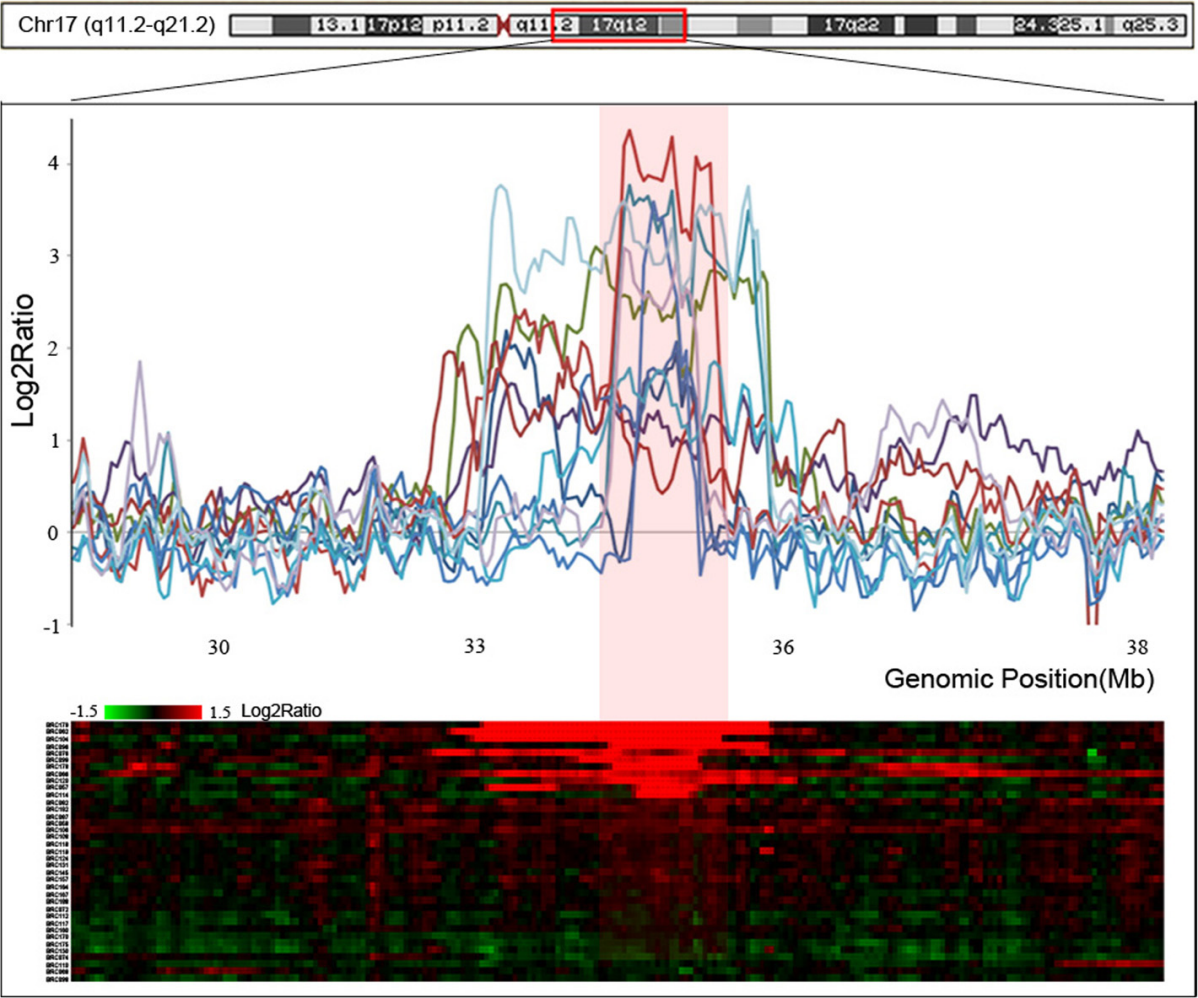
**Example of a recurrently altered region on 17q12.** The bottom plot represents the copy number profile of 48 breast cancers around 7q12 as a heat map. Intensity ratios are plotted in different color scales, reflecting the extent of genomic gains (red) and losses (green), as indicated on the reference color bar. The upper plot illustrates examples of intensity ratios of individual breast cancers. The red zone represents RARs defined using our criteria (frequency > 30% and *P* < 0.05; NEXUS software v3.1). X-axis, genomic position (Mb); Y-axis, Log_2_Ratio.

Except for a few entire chromosomal arm changes, the majority of copy number alterations were regional changes, and some of them were observed repeatedly in the discovery set. Chromosomal segments that covered overlapping CNAs appeared in at least 30% of the samples with *P* < 0.05 and were defined as RARs (RAR-G for gains and RAR-L for losses, respectively). A total of 23 RARs (18 RAR-Gs and 5 RAR-Ls) were defined in the discovery set (Table 2). Figure 2 illustrates the RAR on 17q12 as an example. Of the 23 RARs, 15 RARs detected commonly in both stage I and II groups can be considered earlier events in breast tumorigenesis. Two RARs (RAR-G14 and –G15), which appeared in less than 10% of the stage I samples but in over 40% of the stage II samples, appear to be relatively later events (Table 2). There were six RARs observed in over 50% of the discovery samples: RAR-G2 (1q21.2-q21.3), RAR-G7 (8q24.13), RAR-G8 (8q24.13-21), RAR-G9 (8q24.3), RAR-G10 (8q24.3), and RAR-L1 (8p23.1-p22). All six highly common RARs were earlier events, as described above. Their high prevalence and early occurrence suggest that potential driver cancer genes may be included within the segments. Some cancer-related genes are located in these highly common, early appearing RARs: *MCL1, CTSK, ARNT, S100A10, PTK2, PTP4A3,* and *PSCA* in the RAR-Gs; and *DLC1, PINX1,* and *GATA4* in the RAR-Ls. Many known or putative cancer-related genes are also located in other RARs. For example, the *ERBB2, GRB2,* and *MMP* families, as well as the *TPM3, PYGO2, CKS1B, MUC1, CCT3, PRCC, UBE2C,* and *TNFRSF6B* genes are located in the RAR-Gs while the *PPP2R2A*, *WWOX, TUSC3, MAP2K4,* and *ELAC2* genes are located in the RAR-Ls (Table 2).

**Table 2:** Recurrently altered regions in 48 breast cancer specimens

**RARs**	**Chr**	**Map position**	**Size**	**Cytoband**	***P*- value**	**Freq**	**Freq**	**Freq**	Event*	**Cancer-related genes**
		**(Mp)**	**(Mp)**			**(Total)**	**(S-I)**	**(S-II)**		
RAR-G1	1	143,83-144,30	0.47	q21.1	0.04	48%	36%	51%	Earlier	*TXNIP*
RAR-G2	1	148,12-150,25	2.13	q21.2-q21.3	0.01	50%	36%	54%	Earlier	*MCL1, CTSK, ARNT, SETDB1, SELENBP1, S100A10*
RAR-G3	1	150,75-155,11	4.36	q21.3-q23.1	0.04	48%	36%	51%	Earlier	*CREB3L4, TPM3, HAX1, IL6R, PYGO2, SHC1, CKS1B, NES, ADAM15, EFNA1, MUC1, YY1AP1, ARHGEF2, RAB25, CCT3, CRABP2, HDGF, PRCC*
RAR-G4	1	158,48-159,47	0.99	q23.2-q23.3	0.04	48%	45%	49%	Earlier	*F11R, USF1*
RAR-G5	1	226,10-226,90	0.80	q24.13	0.04	48%	45%	49%	Earlier	*WNT9A, WNT3A*
RAR-G6	6	64,05-64,75	0.70	q12	0.00	38%	45%	35%	Earlier	*-*
RAR-G7	8	123,47-124,74	1.27	q24.13	0.02	50%	36%	54%	Earlier	*FAM83A*
RAR-G8	8	126,23-127,30	1.07	q24.13-q24.21	0.02	50%	36%	54%	Earlier	*-*
RAR-G9	8	140,69-143,86	3.17	q24.3	0.02	50%	36%	54%	Earlier	*PTK2, PTP4A3, PSCA, LYNX1*
RAR-G10	8	144,34-146,27	1.93	q24.3	0.02	50%	36%	54%	Earlier	*SCRIB, HSF1, SLCA4*
RAR-G11	16	0-3,22	3.22	p13.3	0.05	38%	27%	41%	UC	*MPG, AXIN1, RHOT2, STUB1, MSLN, CACNA1H, IGFALS, GFER, TSC2, PDPK1, MMP25*
RAR-G12	16	28,32-31,19	2.87	p11.2	0.05	40%	36%	41%	Earlier	*NUPR1, SULT1A2, SULT1A1, MVP, MAPK3, FUS, PYCARD*
RAR-G13	17	34,97-35,30	0.33	q12	0.03	38%	36%	38%	Earlier	*PPP1R1B, PNMT, PERLD1, ERBB2, GRB7*
RAR-G14	17	70,76-71,21	0.45	q25.1	0.03	38%	9%	46%	Later	*GRB2*
RAR-G15	17	76,90-78,77	1.87	q25.3	0.03	38%	9%	46%	Later	*HGS, SIRT7, RAC3, FASN, CSNK1D*
RAR-G16	20	43,84-44,15	0.31	q13.12	0.00	33%	18%	38%	UC	*UBE2C, MMP9*
RAR-G17	20	60,17-61,61	1.44	q13.33	0.04	31%	18%	35%	UC	*ADRM1, LAMA5, NTSR1, BIRC7, EEF1A2*
RAR-G18	20	61,67-62,44	0.77	q13.33	0.04	31%	27%	32%	UC*	*TNFRSF6B*
RAR-L1	8	8,14-14,07	5.93	p23.1-p22	0.00	50%	36%	54%	Earlier	*CLDN23, PINX1, GATA4, NEIL2, FDFT1, CTSB, DLC1*
RAR-L2	8	15,66-17,12	1.44	p22	0.02	48%	36%	51%	Earlier	*TUSC3, MSR1, FGF20*
RAR-L3	8	24,01-26,72	2.71	p21.2	0.02	48%	36%	51%	Earlier	*ADAM28, GNRH1, PPP2R2A, ADRA1A*
RAR-L4	16	75,79-80,74	4.95	q23.1-q23.3	0.00	33%	27%	35%	UC	*WWOX, DYNLRB2, PLCG2, HSD17B2*
RAR-L5	17	10,54-14,10	3.56	p13.1-p12	0.04	44%	27%	49%	UC	*MAP2K4, ELAC2*

RAR-G: RAR-gain, RAR-L: RAR-loss, Chr: chromosome, Freq: frequency, S-I: stage I, S-II: stage II.

Cancer-related genes: Among all RefSeq genes located in the RARs, the genes which can be searched by the keyword “Cancer/Tumor” from NCBI Gene (http://www.ncbi.nlm.nih.gov/gene) are listed as “cancer-related genes”.

*Earlier: RARs detected commonly in both stage I and II groups, Later: RARs appearing in <10% of stage I samples, but in >40% of stage II samples, UC: unclassified.

### High-level CNAs in EBC

**Figure 3:**
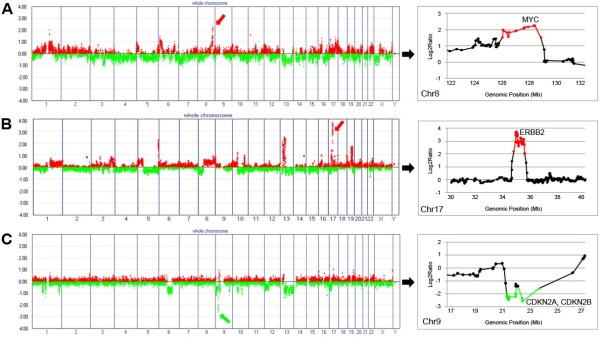
**Examples of high-level copy number alterations.** (**A**) Amplification on 8q24.22, where the *MYC* gene is located (red arrow). (**B**) Amplification on 17q12, where the *ERBB2* gene is located (red arrow). (**C**) Homozygous deletion on 9p21, where *CDKN2A* and *B* are located (red arrow). The red and green spots in the plots on the right represent intensity ratios above 1.5 and below −1.5 on the log_2_ scale, respectively. The X-axis represents individual chromosomes and the Y-axis represents signal intensity ratios (tumor/normal) on the log_2_ scale.

Of the CNAs identified in our EBC specimens, 191 loci were determined to be high-level CNAs (defined as ≥ 1.5 for amplification or ≤ −1.5 for HD on the log_2_ scale), with 158 amplifications and 33 HDs (Additional file 1: Table S3). Of the 158 amplifications, 5 were detected in over 10% of the samples: 1q21.3, 8q24.22, 8q24.3, 16p11.2, and 17q12 (Table 3). These five relatively common amplifications overlapped with earlier event RAR-Gs. Amplification on 17q12, where the *EBBB2* oncogene is located, was the most frequent occurrence (14/48, 29%), followed by amplification on 1q21.3 and 8q24.22 (both 9/48, 19%), where the *LCE* families and *MYC* gene are located, respectively. Examples of high copy number changes are illustrated in Figure 3. There was no HD detected in over 10% of the samples.

**Table 3:** Common high-copy number changes (>10%)

**Changes**	**Chr**	**Position (Mb)**	**Cytoband**	**Size (Mb)**	**Frequency**	**DGV**^**a**^	**Korean CNV**^**b**^	**Putative cancer-related genes**
AMP9	1	150.82-150.93	1q21.3	0.11	9 (19%)	Y	Y	*LCE3C, LCE3B, LCE3A, LCE2D, LCE2C, LCE2B*
AMP64	8	120.51-135-87	8q24.22	15.37	9 (19%)	Y	Y	*DDEF1, ENPP2, FAM83A, FAM84B, HAS2, MTSS1, MYC, NDRG1, RNF139, TG, WISP1*
AMP67	8	144.37-145.55	8q24.3	1.18	5 (10%)	Y	Y	*SCRIB, PLEC1, HSF1*
AMP113	16	28.40-28.42	16p11.2	0.02	6 (13%)	Y	N	*CLN3*
AMP119	17	32.42-35.87	17q12	3.45	14 (29%)	Y	N	*AATF, ACACA, CDC6, CSF3, ERBB2, FBXO47, GRB7, IGFBP4, LASP1, PCGF2, PERLD1, PLXDC1, PNMT, PPP1R1B, RARA, RPL19, RPL23, TBC1D3, THRA, TOP2A*

^a^ Overlapping status with DGV (http://projects.tcag.ca/variation/) entries; ^b^ Overlapping status with CNVs identified from Koreans [35].

Y: overlap, N: no overlap, AMP: amplification, Chr: chromosome.

### Association of RARs with clinicopathologic features

Five clinical variables (age at diagnosis, stage, ER status, PR status, and HER2 status) were analyzed to assess their association with RARs (see Additional file 1). Only RAR-L4 was significantly associated with ER-positivity (*P* < 0.0001, FDR-corrected P = 0.015) (Additional file 1: Table S4). When we observed the distribution of the RARs by molecular subtypes (Luminal A, Luminal B, HER2 and TNBC), only RAR-G13 was differently distributed among the subtypes (*P* = 1.77 × 10^-4^) (Additional file 1: Table S5).

### RARs associated with prognosis in EBC

**Figure 4:**
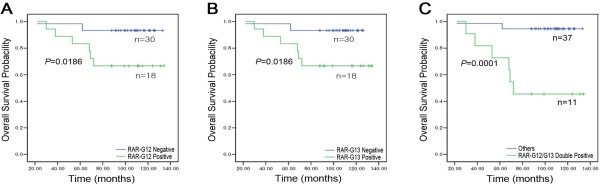
**Kaplan–Meier curves by RAR status in the discovery set.** (**A**) RAR-G12 (16p11.2) positives *vs.* negatives, (**B**) RAR-G13 (17q12) positives *vs*. negatives, and (**C**) RAR-G12/G13 double-positives *vs*. others. The curves show overall survival.

Univariate survival analysis was performed to screen RARs that have potential implications on patient survival. Univariate analysis was also performed to identify clinicopathologic features (age at diagnosis, stage, ER, PR, and HER2) for inclusion as covariates for Cox regression. The event was defined as a death within ten years of the diagnosis. Among the genetic and clinical factors, RAR-G12 (16p11.2) and RAR-G13 (17q12) were significantly associated with poor survival in the discovery set of 48 EBCs (*P* = 0.0186 and *P* = 0.0186, respectively) (Figures 4A and B).

The role of combinations of the 23 RARs and their association with poor survival was also explored, as described in the Methods. As a result, eight combination rules were found to be potentially associated with death events (Laplace accuracy score >0.75) (Additional file 1: Table S6). All eight poor survival-associated rules contained ‘RAR-G12 and RAR-G13 positives,’ suggesting that the co-occurrence of these two alterations may affect EBC prognosis. Indeed, on univariate survival analysis, RAR-G12 and −13 double-positives had a significantly worse prognosis compared with the prognosis of others (*P* = 0.0001) (Figure 4C). Among the clinicopathologic features, none of them was associated with survival.

### Replication of the poor prognosis-associated RARs

**Figure 5:**
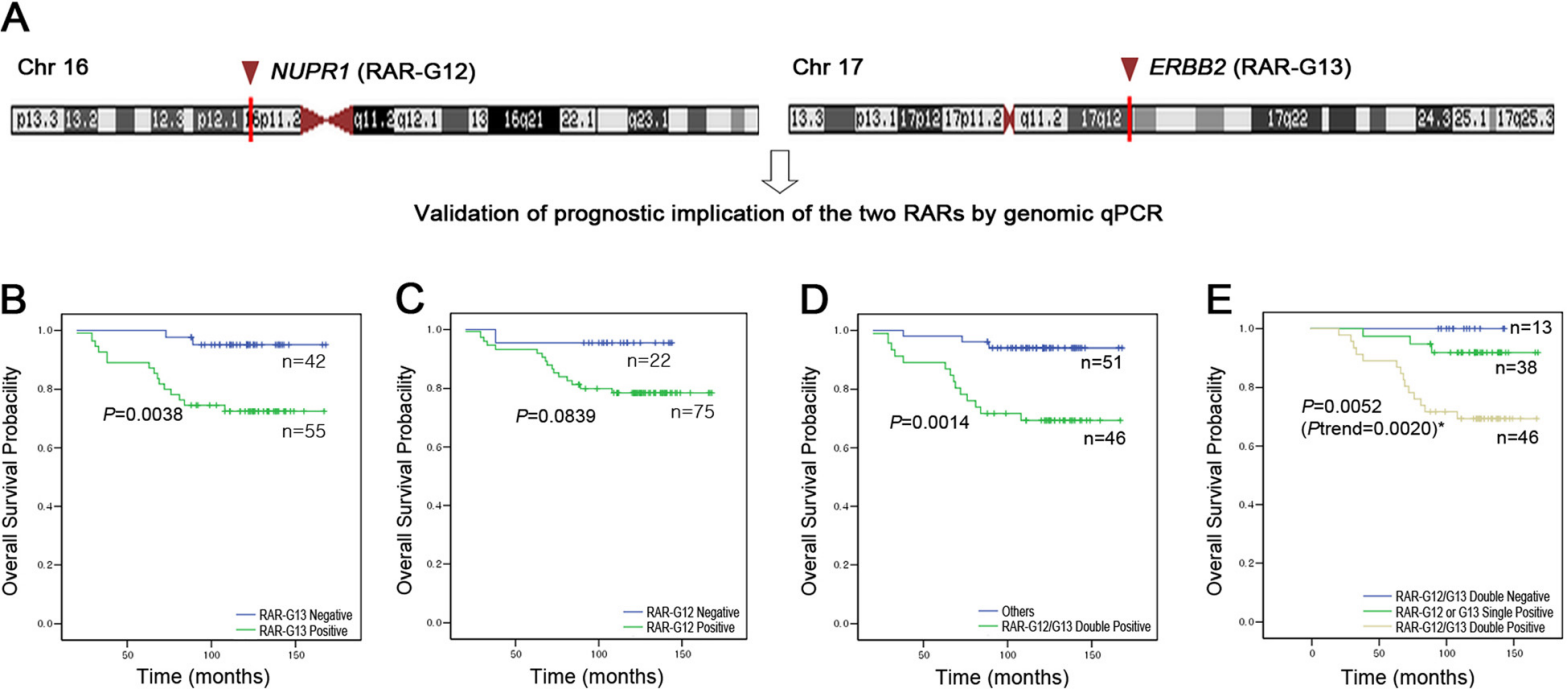
**Kaplan–Meier curves by RAR status in the replication set.** (**A**) Genomic qPCR system targets; *NUPR1* gene located in RAR-G12; *ERBB2* gene located in RAR-G13. (**B**) *NUPR1* (RAR-G12) positives *vs*. negatives. (**C**) *ERBB2* (RAR-G13) positives *vs*. negatives. (**D**) *NUPR1-ERBB2* double-positives *vs*. others. (**E**) Survival curves by subgroup; RAR-G12/13 double-positives, positive for either RAR-G12 or G-13, and double-negatives. The curves show overall survival.

In order to validate the prognosis-associated RARs identified in the discovery set, a genomic qPCR system was designed to target the *NUPR1* gene located in RAR-G12 and the *ERBB2* gene located in RAR-G13 (Figure 5A). For independent validation, the DNA extracted from FFPE samples of 97 EBCs (replication set) was used. On univariate survival analysis of the replication set, patients positive for RAR-G13 (*ERBB2*) were found to have a significantly poorer survival than patients without the *ERBB2* gain (*P* = 0.0038) (Figure 5B). Patients positive for RAR-G12 (*NUPR1*) also showed poorer survival than negative patients, but only with borderline significance (*P* = 0.0839) (Figure 5C). As expected, the patients positive for both *NUPR1* and *ERBB2* gains had a significantly poorer prognosis than patients in the replication set that did not have this combination (*P* = 0.0014) (Figure 5D). When survival curves were compared based on a more detailed RAR status (positive for both RAR-G12 and −13, positive for either RAR-G12 or −13, and negative for both), the survival probabilities among the three groups were significantly different, with the probability of surviving decreasing with greater RAR positivity (*P* = 0.0052; *P* for trend = 0.0020) (Figure 5E).

Multivariate analysis with the two significant RARs identified on univariate analysis and the covariates (age at diagnosis, stage, ER status, PR status, and HER2 status) revealed that the copy number gain status of *ERBB2* (RAR-G13) was an independent indicator of poor prognosis in EBC (Hazard ratio [HR] = 5.36 , 95% CI 1.80-15.98, *P* = 0.003) (Table 4). When the multivariate analysis was performed with *NUPR1-ERBB2* combined status and the same covariates, positivity for both RAR-Gs was found to be a strong independent indicator of poor prognosis, showing additive effects (HR = 7.31, 95% CI 2.65-20.15, *P* = 0.0001) (Table 4).

**Table 4:** Results of multivariate aalysis

**Variable**	**Hazard ratio**	**95% confidence interval**		*P*value*
		**Lower**	**Upper**	
**Using each RAR**				
Age	1.01	0.41	2.48	0.986
Stage	3.54	1.01	12.39	0.048
ER	1.63	0.61	4.34	0.325
PR	0.82	0.32	2.13	0.690
HER2	0.48	0.19	1.17	0.105
RAR-G12	2.55	0.73	8.90	0.141
RAR-G13	5.36	1.80	15.98	**0.003**
**Using RAR double-positives**				
Age	1.00	0.41	2.44	0.993
Stage	3.73	1.06	13.04	**0.040**
ER	1.60	0.61	4.24	0.343
PR	0.84	0.32	2.15	0.709
HER2	0.46	0.19	1.14	0.094
RAR-G12*RAR-G13	7.31	2.65	20.15	**0.0001**

RAR-G12*RAR-G13: *NUPR1* (RAR-G12) and *ERBB2* (RAR-G13) copy number gain double-positives.

*Significant result (*P* < 0.05).

## Discussion

In this study, we analyzed the genome-wide copy number alteration profiles in 48 EBCs using 30K oligoarray-CGH. We delineated RARs under the assumption that commonly altered chromosomal segments in EBCs may contain driver genes essential for initiation or early progression of breast tumorigenesis. It is also possible that some RARs have prognostic implications in EBC. To explore this possibility, we defined RARs in a discovery set of EBC and examined their associations with prognosis. A total of 23 RARs were defined, and all of them were found to overlap at least one of the recently reported CNAs in breast cancer including EBC, suggesting the reliability of our data [14-17]. The nature of RARs (gain or loss) was also largely consistent with the previous observations. For example, RAR-L3 (8p21.2) and RAR-L5 (17p12), where *PPP2R2A* and *MAP2K4* are located, respectively, and RAR-G13 (17q12), where *ERBB2* is located, were consistently detected in a recent large-scale breast cancer genetic subgroup study [14]. In particular, 21 of the 23 RARs overlap recurrent copy number alterations identified in EBCs (stage I and II) from whites, blacks, and Hispanics by Thompson et al.’s recent study [15]. However, the recurrent gain on 14q11.2 in Thompson et al.’s report was not detected in our array-CGH analysis. This difference, which requires further investigation, may be due to a Korean EBC-specific feature or to the probe design of the array-platform used in this study. We validated the association of RARs with prognosis in the larger independent replication set of 97 EBCs. In addition to RARs, some entire chromosomal arm changes were also commonly observed (> 30% of the samples) in this study (Additional file 1: Table S7), and are largely consistent with previous observations in breast cancer of diverse ethnic groups [11,25].

Of the RARs identified in this study, 15 were commonly detected in both stages I and II, which suggests that these copy number alterations were acquired at an earlier stage of EBC. In particular, 6 of the 15 earlier event RARs, RAR-G2 (1q21.2-q21.3), RAR-G7 (8q24.13), RAR-G8 (8q24.13-21), RAR-G9 (8q24.3), RAR-G10 (8q24.3), and RAR-L1 (8p23.1-p22), appeared in over 50% of cases. Some genes located in these six RARs have been suggested to be involved in early breast tumorigenesis. For instance, the *PTK2* gene located on 8q24.3 (RAR-G9) is a member of the focal adhesion kinase (FAK) subfamily of protein tyrosine kinases. Overexpression of FAK was suggested to be an early event in DCIS tumorigenesis [26]. Although the protein levels of potential cancer-related genes in these six highly common loci were not examined in this study, our data suggest that the six alterations may be commonly occurring genetic events in the initial stage of breast cancer development. Based on our findings, two RARs on 17q25 can be considered relatively late events in breast tumorigenesis, since the RARs on 17q25 (RAR-G14 and -G15) were scarcely observed in stage I (<10%), but were quite frequent (>45%) in stage II. Interestingly, a copy number gain on 17q25.3 was reported to be one of the recurrence-associated chromosomal alterations in one previous report on Korean women with breast cancer [27].

When we assessed the prognostic implications of RARs, RAR-G12 (16p11.2) and RAR-G13 (17q12) were significantly associated with poorer prognosis in the discovery set. A number of cancer-related genes are located in these two RARs: *NUPR1, MVP, MAPK3, FUS,* and *PYCARD* are located in RAR-G12 while *ERBB2*, *GRB7,* and *PPP1R1B* are located in RAR-G13. Among these potential cancer-related genes, Nupr1 is known to interact with various molecules involved in cell cycle regulation, programmed cell death and transcription activity. For these reasons, Nupr1 is a potential molecular target in the development of anticancer drugs [28]. Although the *NUPR1* gene has been suggested to be responsible for the growth and progression of many cancers including breast cancer [29,30], the prognostic implications of the *NUPR1* gene in EBC have not been reported. Amplification and overexpression of the *ERBB2* oncogene in RAR-G13 (17q12) is known to be associated with high recurrence rates and reduced breast cancer survival [31-33]. The frequent copy number gains (38%) and amplification (29%) of *ERBB2* in this study are consistent with previous studies on breast cancer [11,34].

In a replication analysis by genomic qPCR, the prognostic implication of *ERBB2* gain (RAR-G13) was successfully replicated in the larger replication set, but that of the *NUPR1* gain (RAR-G12) was not. We hypothesized that the *NUPR1* gain itself might not be an influential alteration, but that EBC prognosis is more strongly affected by the co-occurrence of *NUPR1* with a strong driver mutation (*ERBB2*). Association-rule mining results also supported the predictive power of their co-occurrence for poor prognosis. As expected, when these two RARs were combined and used as an independent factor, the hazard ratio increased in an additive manner. A stronger significance level was also achieved on Cox regression analysis compared with when only *ERBB2* was used, which may reflect the multigenic nature of cancer.

In this study, 191 high-level CNAs (158 amplifications and 33 HDs) were detected by array-CGH, and 5 of them were detected in more than 10% of the samples. A substantial number of the high-level CNAs overlap database of genomic variants (DGV, http://projects.tcag.ca/variation/) entries and the copy number variants (CNVs) identified from Koreans [35]. Although the limitations of DGV are well known in terms of accuracy and overestimation, we cannot rule out the possibility that some high-level CNAs identified in this study are copy CNVs because we used DNA from a single individual as a universal reference. All five of the common amplifications (observed in >10% of the samples) also overlap the CNV loci in DGV. However, four of them, except for one very small (0.02 Mb) amplification on 16p11.2, were reported to be amplifications or copy number gains in breast cancer by a recent high-resolution array-CGH analysis [15-17,36], suggesting that these four common amplifications are likely CNAs. The amplification frequency of *ERBB2* in this study was largely similar to the previous studies including Koreans [37-39].

There are several limitations in this study. First, due to the limited sample size of subtypes, we could not see the prognostic implications of the RARs in the four molecular subtypes properly. Second, we did not examine the molecular mechanisms of the synergistic effect of the *ERBB2-NUPR1* co-occurrence. Further studies will be required to delineate the roles of NUPR1 gain and the simultaneous *ERBB2-NUPR1* gains in early breast tumorigenesis. Third, we used single reference DNA in this study, so it is possible that some of the CNAs identified in this study are CNVs, especially small-sized CNAs overlapping previously reported CNVs.

## Conclusion

In this study, we found six highly common RARs in EBCs and determined the potential of simultaneous alterations of *ERBB2* (17q12) and *NUPR1* (16p11.2) as significant predictors of poor prognosis in EBC. Our study will help to elucidate the molecular mechanisms underlying early-stage tumorigenesis in breast cancer. In addition, our study shows the potential for combinations of copy number alterations to be used as prognosis predictors for early-stage breast cancer.

## Abbreviations

EBC: Early-stage breast cancer; CNA: Copy number alteration; RAR: Recurrently altered region; array-CGH: Oligoarray-comparative genomic hybridization.

## Competing interests

The authors declare that they have no competing interests.

## Authors’ contribution

SHJ executed most experiments and drafted the manuscript. AWL collected the patient specimens and was involved in data analysis. SHY participated in the design of this study, performed statistical analysis and wrote the manuscript. HJH performed an association rule mining and drafted the manuscript. CC was involved in data analysis and preparing the figures. YJC proposed this study, organized the research team, interpreted all the data, and participated in writing the manuscript. All authors read and approved the final manuscript.

## Pre-publication history

The pre-publication history for this paper can be accessed here:

http://www.biomedcentral.com/1471-2407/12/382/prepub

## Supplementary Material

Additional file 1 **Figure S1.** Genome-wide frequency plots of chromosomal alterations for each breast cancer subtype. **Table S1.** Primer sequences for target and diploid control regions. **Table S2.** Copy number alterations significantly more frequent in stage II than in stage I. **Table S3.** High copy number changes in the discovery set of early breast cancers. **Table S4.** Correlation between RARs and clinicopathologic features. **Table S5.** RARs in 48 breast cancers by molecular subtype. **Table S6.** RAR combination rules associated with death events. **Table S7.** Frequency of chromosomal arm changes in 48 breast cancers. Click here for file
