# Supplementary material for: Simultaneous copy number gains of NUPR1 and ERBB2 predicting poor prognosis in early-stage breast cancer
Source: BMC Cancer. 2012 Aug 31;12:382. doi: 10.1186/1471-2407-12-382 (PMC3489802; doi:10.1186/1471-2407-12-382)
Supplement: Additional file 1 — Figure S1. Genome-wide frequency plots of chromosomal alterations for each breast cancer subtype. Table S1. Primer sequences for target and diploid control regions. Table S2. Copy number alterations significantly more frequent in stage II than in stage I. Table S3. High copy number changes in the discovery set of early breast cancers. Table S4. Correlation between RARs and clinicopathologic features. Table S5. RARs in 48 breast cancers by molecular subtype. Table S6. RAR combination rules associated with death events. Table S7. Frequency of chromosomal arm changes in 48 breast cancers. [file 1471-2407-12-382-S1.pdf]

Additional Data

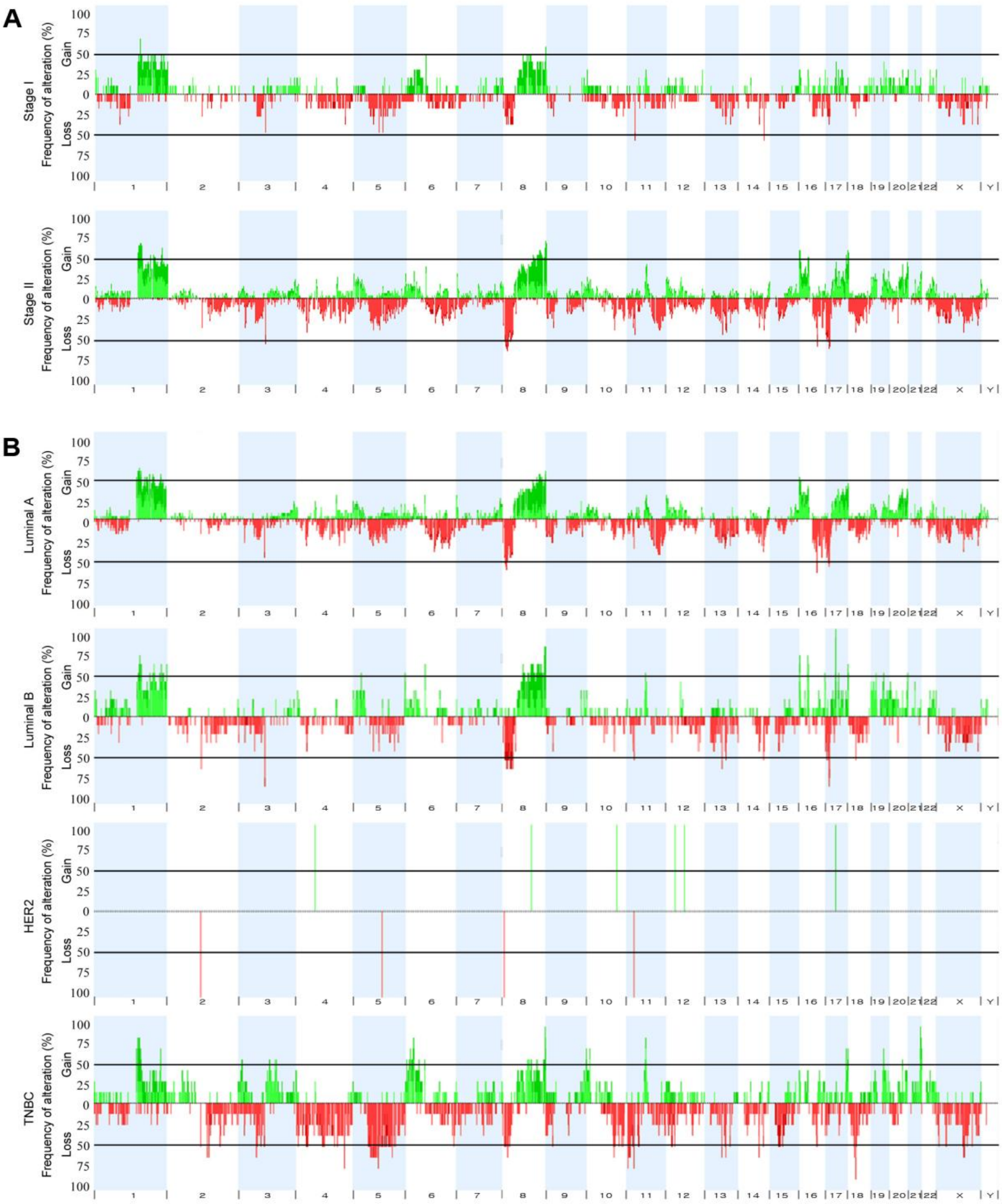

Figure S1. Genome-wide frequency plots of chromosomal alterations for each breast cancer subtype.
(A) Frequencies of copy number gains and losses in Stage I (n=11) and II (n-37) breast cancers.
(B) Frequencies of copy number gains and losses in Luminal A (n=29), Luminal B (n=10), HER2 (n=1), and TNBC (n=8). Green bars denote copy number gains and red bars copy number losses. Boundaries of individual chromosomes are indicated by the vertical bars.

Table S1. Primer sequences for target and diploid control regions

Target Region	Forward Primer (5' – 3')	Reverse Primer (5' – 3')
RAR-G12 (*NUPR1*)	AGCTTCTCTCTTGGTGCGACCTTT	ATCATGCCTATGCCCACTTCACCT
RAR-G13 (*ERBB2*)	TGAGGGCAGTTACCAGTGCCAATA	ATCGTGCCCACTCTTACCCATCAA
Diploid control (13q32.1)	CGCTACCACCACACCAAGCAG	CCACCTGGCTGTTGTAGTCCTC

Table S2. Copy number alterations significantly more frequent in stage II than in stage I

Region (Mb)	Cytoband	Event	Frequency (Stage I, %)	Frequency (Stage II, %)	*P*-Value	FDR
chr16:3.61-3.65	p13.3	Gain	0	37.8	0.021	1
chr16:3.65-4.21	p13.3	Gain	0	35.1	0.023	1
chr16:19.12-19.62	p12.3	Gain	0	32.4	0.044	1
chr16:19.62-20.42	p12.3	Gain	0	35.1	0.023	1
chr16:20.42-20.58	p12.3 - p12.2	Gain	0	32.4	0.044	1
chr16:20.58-20.65	p12.2	Gain	0	29.7	0.048	1
chr16:27.36-27.72	p12.1 - p11.2	Gain	0	29.7	0.048	1
chr16:60.23-60.24	q21	Loss	18.2	56.8	0.039	1
chr17:0.62-0.64	p13.3	Loss	0	40.5	0.01	1
chr17:0.64-0.70	p13.3	Loss	0	37.8	0.021	1
chr17:0.70-0.80	p13.3	Loss	0	35.1	0.023	1
chr17:15.07-15.33	p12	Loss	0	32.4	0.044	1
chr17:15.33-15.34	p12	Loss	0	29.7	0.048	1
chr17:70.75-71.18	q25.1	Gain	9.1	45.9	0.035	1
chr17:71.18-71.20	q25.1	Gain	9.1	48.6	0.032	1
chr17:71.20-71.21	q25.1	Gain	9.1	45.9	0.035	1
chr17:71.23-71.32	q25.1	Gain	0	43.2	0.009	1
chr17:71.32-71.85	q25.1	Gain	0	40.5	0.01	1
chr17:76.00-76.57	q25.3	Gain	0	35.1	0.023	1
chr17:76.87-76.89	q25.3	Gain	9.1	45.9	0.035	1
chr17:76.89-76.93	q25.3	Gain	9.1	48.6	0.032	1
chr17:76.93-77.01	q25.3	Gain	9.1	51.4	0.016	1
chr17:77.01-77.08	q25.3	Gain	18.2	54.1	0.045	1
chr17:77.44-77.58	q25.3	Gain	9.1	45.9	0.035	1
chr17:77.58-77.59	q25.3	Gain	9.1	48.6	0.032	1
chr17:77.59-78.20	q25.3	Gain	9.1	51.4	0.016	1
chr17:78.20-78.24	q25.3	Gain	9.1	48.6	0.032	1
chr17:78.24-78.77	q25.3	Gain	9.1	51.4	0.016	1
chr20:28.12-28.13	q11.1	Loss	0	29.7	0.048	1

Table S3. High copy number changes in the discovery set of early breast cancers

Changes	Cytoband	Portion (Mb)	Size (Mb)	Number of case	Observed cases	Putative cancer-related genes	DGV*	Korean CNV †
Amp1	1p36.33	1.18-1.26	0.08	1	BRC104		yes	no
Amp2	1p35.2-p34	31.75-32.15	0.40	1	BRC62		yes	no
Amp3	1p34.2-p33	43.22-44.24	1.02	1	BRC62	MPL, CDC20, PTPRF, ST3GAL3, ATP6V0B, B4GALT2	yes	yes
Amp4	1p33	50.20-50.71	0.51	1	BRC104		yes	yes
Am p5	1p12-p11.2	119.63-120.96	1.33	2	BRC88, 104	HSD3B2, HSD3B1, REG4, NOTCH2	yes	yes
Amp6	1q21.1	144.15-144.40	0.25	1	BRC110	TXNIP, PIAS3	yes	yes
Amp7	1q21.2	148.40-149.41	1.01	1	BRC98	PLEKHO1, ARNT, CTSK, LASS2, MCL1, PRUNE, SETDB1	yes	no
Amp8	1q21.3	150.01-150.05	0.04	1	BRC110		yes	no
Amp9	1q21.3	150.82-150.93	0.11	9	BRC98, 104, 117, 130, 145, 157, 175, 182, 185		yes	yes
Amp10	1q21.3	152.45-152.51	0.07	1	BRC110		yes	no
Amp11	1q21.3	153.16-153.31	0.14	1	BRC98	PYGO2, SHC1, CKS1B, ADAM15	yes	no
Amp12	1q22	153.50-154.31	0.81	1	BRC98	FDPS, YY1AP1, DAP3, ARHGEF2, RAB25	yes	no
Amp13	1q22	154.53-155.05	0.52	1	BRC98	VHLL, MEF2D, NES, CRABP2, PRCC, CCT3	yes	no
Amp14	1q23.1	156.42-157.18	0.76	1	BRC110	CD1D, PYHIN1	yes	yes
Amp15	1q23.3	159.40-159.41	0.01	1	BRC106		yes	no
Amp16	1q23.3	160.61-160.76	0.15	1	BRC110		yes	no
Amp17	1q24.1	165.09-165.54	0.45	1	BRC110	POU2F1	yes	no
Amp18	1q24.2	167.03-167.75	0.72	1	BRC110		yes	yes
Amp19	1q32.2	206.03-206.27	0.24	1	BRC148	CD46, CD34	yes	no
Amp20	1q32.2	208.03-208.41	0.38	1	BRC148		yes	yes
Amp21	1q42.12	223.16-223.40	0.24	1	BRC98		yes	no
Amp22	1q42.12	224.34-224.98	0.64	1	BRC98	PARP1	yes	no
Amp23	1q42.13	225.95-226.44	0.48	2	BRC98, 179	WNT9A, WNT3A, ARF1	yes	no
Amp24	1q42.2	227.86-229.54	1.67	2	BRC88, 179	AGT, CAPN9	yes	no
Amp25	1q42.2	229.56-231.19	1.63	1	BRC88	EGLN1	yes	yes
Amp26	1q43	231.87-235.13	3.25	1	BRC88	ARID4B, B3GALNT2, NID1, LGALS8, MTR	yes	yes
Amp27	1q43	236.12-239.57	3.46	1	BRC88	RGS7	yes	yes
Amp28	1q43	239.74-241.73	1.99	1	BRC88	FH, EXO1, SDCCAG8, AKT3	yes	no
Amp29	1q44	242.30-244.09	1.79	1	BRC88	SMYD3	yes	no
Amp30	1q44	245.04-247.07	2.03	1	BRC88		yes	yes
Amp31	2p21	42.01-42.51	0.49	1	BRC87	COX7A2L	yes	no
Amp32	2p21p	46.27-46.84	0.58	1	BRC87	PRKCE, EPAS1	yes	yes
Amp33	2p16.3	48.46-48.86	0.40	1	BRC87	LHCGR	yes	yes
Amp34	2q11.2	95.18-96.84	1.66	1	BRC110	STARD7	yes	yes
Amp35	2q24.2	160.12-160.37	0.25	1	BRC87		yes	no
Amp36	2q33.1	196.31-200.89	4.58	1	BRC87	HSPD1, HSPE1	yes	yes
Amp37	2q33.3	206.73-206.88	0.15	2	BRC96, 131		yes	no
Amp38	5p15.3	0.21-1.99	1.78	1	BRC74	AHRR, SLC9A3, TPPP, ZDHHC11, SLC12A7, TERT	no	yes
Amp39	5p15.33	3.47-3.65	0.18	1	BRRC74		no	no
Amp40	5p13.2	37.10-37.26	0.16	1	BRC120	NIPBL	no	no
Amp41	5q35.3	175.70-176.97	1.26	2	BRC96, 104	ARL10, UNC5A, FGFR4, RAB24	yes	no
Amp42	6p21.3	26.13-26.14	0.01	1	BRC189		yes	no

Amp43	6p21.3	26.21-26.35	0.14	1	BRC189		yes	no
Amp44	6q21	106.66-112.78	6.11	1	BRC120	AMD1, CD164, FYN, LAMA4, PDSS2, PRDM1, TRAF3IP2, WISP3	yes	yes
Amp45	7p14.3	32.87-33.80	0.93	1	BRC74		no	yes
Amp46	7p14.2	35.64-35.84	0.20	1	BRC74		yes	no
Amp47	7q32.2	129.21-129.56	0.35	1	BRC110		yes	no
Amp48	8p23.1	10.38-10.57	0.19	1	BRC120		yes	no
Amp49	8p11.2	36.91-40.15	3.24	2	BRC66, 74	GPR124, ADRB3, EIF4EBP1, LSM1, BAG4, FGFR1, TACC1, ADAM9, INDO	yes	yes
Amp50	8q11.23	55.54-55.71	0.16	1	BRC157		yes	no
Amp51	8q12.1	56.10-57.29	1.19	1	BRC76	LYN, PLAG1	yes	no
Amp52	8q12.3	64.10-64.46	0.36	2	BRC76, 157		yes	no
Amp53	8q13	66.80-67.25	0.45	1	BRC82	CRH	yes	no
Amp54	8q13	67.75-68.04	0.28	1	BRC82	PTTG3	yes	no
Amp55	8q21.11	73.45-79.74	6.30	2	BRC76, 189	TERF1, TCEB1	yes	yes
Amp56	8q21.13	80.99-86.31	5.32	2	BRC76, 189	TPD52, E2F5	yes	yes
Amp57	8q21.3	87.13-92.16	5.03	1	BRC76	WWP1, MMP16, NBN	yes	yes
Amp58	8q22	94.80-97.31	2.52	3	BRC76, 179, 189	CDH17, RAD54B, CCNE2, TP53INP1	yes	yes
Amp59	8q22	97.69-99.20	1.51	3	BRC76, 179, 189	SDC2, MTDH, LAPTM4B	yes	yes
Amp60	8q23.1	101.78-108.33	6.56	6	BRC76, 82, 88, 98, 179, 189	PABPC1, YWHAZ, RRM2B, KLF10, ATP6V1C1, CTHRC1, RIMS2, LRP12, ANGPT1	yes	yes
Amp61	8q23.3	109.34-113.30	3.96	2	BRC76, 189	NUDCD1, EBAG9	yes	yes
Amp62	8q24.11	116.49-117.93	1.44	1	BRC189	TRPS1, RAD21	yes	yes
Amp63	8q24.11	119.17-119.38	0.22	1	BRC76	EXT1	yes	yes
Amp64	8q24.22	120.51-135-87	15.37	9	BRC76, 82, 88, 104, 110, 130, 157, 182, 189	DDEF1, ENPP2, FAM83A, FAM84B, HAS2, MTSS1, MYC, NDRG1, RNF139, TG, WISP1	yes	yes
Amp65	8q24.23	139.22-139.52	0.29	1	BRC82		yes	no
Amp66	8q24.3	142.30-143.85	1.55	4	BRC82, 117, 120, 157	PTP4A3, BAI1, ARC, PSCA, LY6K, LYNX1	yes	no
Amp67	8q24.3	144.37-145.55	1.18	5	BRC82, 117, 120, 157, 189	SCRIB, PLEC1, HSF1	yes	yes
Amp68	8q24.3	145.59-145.72	0.13	2	BRC82, 157	SLC39A4, VPS28	yes	no
Amp69	8q24.3	145.92-146.04	0.12	2	BRC82, 157		yes	no
Amp70	9p24	2.41-2.64	0.23	1	BRC189		yes	yes
Amp71	9q13	70.33-71.24	0.90	1	BRC104	FXN	yes	yes
Amp72	9q33.2	123.10-124.13	1.02	1	BRC110	GSN, DAB2IP, LHX6	yes	no
Amp73	9q33.2	124.42-124.66	0.24	1	BRC110		yes	no
Amp74	9q33.3	125.23-126.07	0.85	1	BRC110		yes	yes
Amp75	9q33.3	126.28-126.80	0.51	1	BRC110		yes	no
Amp76	9q33.3	127.04-128.64	1.60	1	BRC110	HSPA5	yes	yes
Amp77	10q11.21	43.46-43.80	0.34	1	BRC64		no	no
Amp78	10q22.1	72.31-72.79	0.48	1	BRC110	UNC5B	yes	no
Amp79	10q22.2	76.87-76.92	0.05	1	BRC110		yes	no
Amp80	10q22.3	79.78-80.75	0.96	1	BRC148		yes	no
Amp81	11p13	31.39-32.08	0.69	1	BRC106		no	yes
Amp82	11q12.2	59.33-60.32	1.00	1	BRC74	MS4A1	yes	yes
Amp83	11q13	67.69-68.14	0.45	1	BRC106		yes	no
Amp84	11q13.3	68.5-69.99	1.49	3	BRC76, 99, 106	MYEOV, CCND1, ORAOV1, FGF19, FGF4, FGF3, TMEM16A, FADD, CTTN	yes	no
Amp85	11q13.4	70.96-71.39	0.43	2	BRC57, 58	NUMA1	yes	no
Amp86	11q13.5	72.62-72.69	0.07	1	BRC106	P2RY2, P2RY6	yes	no
Amp87	11q14.1	73.25-82.12	8.87	1	BRC74, 106	ARRB1, CHRDL2, NEU3, PAK1, RPS3, RSF1,	yes	yes

						THRSP, UVRAG		
Amp88	11q14.1	82.63-85.08	2.45	1	BRC106	SYTL2	yes	yes
Amp89	12p13	6.73-6.76	0.03	1	BRC130	PTMS	yes	no
Amp90	12p13.2	10.11-10.2	0.09	1	BRC148		yes	yes
Amp91	12p11.21	28.63-31.07	2.44	1	BRC148	ERGIC2	yes	yes
Amp92	12q12	44.11-44.40	0.29	1	BRC88		yes	no
Amp93	12q14.1	62.87-63.18	0.31	1	BRC74	TBK1	yes	no
Amp94	12q14.3	64.14-64.51	0.36	1	BRC74	HMGA2	yes	yes
Amp95	12q21.1	66.34-70.71	4.37	1	BRC74	IFNG, SLC35E3, MDM2, YEATS4, PTPRB, TSPAN8, LGR5	yes	yes
Amp96	13q12	22.45-23.20	0.75	1	BRC96		no	no
Amp97	13q12.2	26.23-27.77	1.55	1	BRC96	RASL11A, CDX2, FLT3, FLT1	no	yes
Amp98	13q13.1	31.87-31.98	0.10	1	BRC96		no	no
Amp99	13q14.11	37.11-44.41	7.30	1	BRC96	TNFSF11, DNAJC15, TSC22D1	no	yes
Amp100	13q14.3	50.02-51.89	1.87	1	BRC178	INTS6, NEK3	no	yes
Amp101	13q22.1	73.73-74.03	0.30	1	BRC178		no	yes
Amp102	13q31.1	78.83-79.03	0.20	1	BRC178		no	no
Amp103	14q23.3	62.72-74.47	1.75	1	BRC114	ESR2, HSPA2	no	yes
Amp104	14q31.3	87.67-87.72	0.04	1	BRC96		no	no
Amp105	14q32	100.27-100.37	0.10	1	BRC188	MEG3	no	no
Amp106	15q24.1	71.82-71.97	0.15	3	BRC66, 130, 145		no	no
Amp107	16p13.3	0.56-0.71	0.16	3	BRC82, 104, 157	STUB1, RHOT2	yes	no
Amp108	16p13.3	1.76-1.77	0.01	1	BRC82		yes	no
Amp109	16p13.3	2.50-2.61	0.11	1	BRC157	PDPK1	yes	yes
Amp110	16p13.3	2.84-2.94	0.10	1	BRC82		yes	no
Amp111	16p13.13	11.25-11.68	0.43	1	BRC104	SOCS1, PRM2, LITAF	yes	yes
Amp112	16p13.1	15.59-16.16	0.58	1	BRC104	ABCC1	yes	yes
Amp113	16p11	28.40-28.42	0.02	6	BRC64, 74, 82, 96, 157, 179		yes	no
Amp114	16q22.1	69.27-69.32	0.05	1	BRC64		yes	no
Amp115	16q22.1	70.62-70.70	0.08	1	BRC64	HP	yes	no
Amp116	16q22.3	71.20-71.38	0.18	1	BRC64		yes	no
Amp117	17p11.2	19.18-20.78	1.60	1	BRC120	MAPK7	yes	no
Amp118	17p11.2	27.72-27.75	0.03	1	BRC178		no	no
Amp119	17q21.2	32.42-35.87	3.45	14	BRC57, 59, 62, 64, 66, 76, 96, 99, 104, 114, 120, 178, 179, 189	AATF, ACACA, CDC6, CSF3, ERBB2, FBXO47, GRB7, IGFBP4, LASP1, PCGF2, PERLD1, PLXDC1, PNMT, PPP1R1B, RARA, RPL19, RPL23, TBC1D3, THRA, TOP2A	yes	no
Amp120	17q21.2	36.99-37.26	0.27	1	BRC64	KRT17, GAST, JUP	yes	yes
Amp121	17q21.31	38.53-38.71	0.18	1	BRC178	NBR2, NBR1	yes	no
Amp122	17q21.33	44.40-45.91	1.51	2	BRC178, 189	IGF2BP1, ABI3, PHB, NGFR, ITGA3, COL1A1	yes	no
Amp123	17q23.2	54.42-55.88	1.46	1	BRC64	TRIM37, RPS6KB1, APPBP2	yes	yes
Amp124	17q24.1	57.38-60.21	2.83	2	BRC64, 74	ACE, GH1, ERN1, PECAM1, DDX5, ICAM2	yes	yes
Amp125	17q24.2	63.42-64.05	0.63	1	BRC74	KPNA2, PRKAR1A	no	yes
Amp126	17q25.1	69.95-71.31	1.36	2	BRC178, 189	SLC9A3R1, GRB2, ITGB4	yes	no
Amp127	17q25.2	71.90-72.24	0.34	1	BRC189	CYGB, ST6GALNAC1	yes	no
Amp128	17q25.3	73.67-74.36	0.69	1	BRC189	TK1, BIRC5, EPR1, SOCS3, TIMP2	yes	no
Amp129	18q12.3	41.52-41.73	0.22	1	BRC98		yes	no
Amp130	18q22.2	64.87-65.66	0.79	8	BRC58, 88, 117, 118, 133, 175, 185, 188		yes	yes
Amp131	19p13.3	0.85-0.92	0.08	2	BRC82, 148	KISS1R	no	no

Amp132	19p13.3	1.73-1.80	0.07	2	BRC96, 157		no	no
Amp133	19p13.3	1.93-1.94	0.01	2	BRC157, 189		no	no
Amp134	19q12	34.19-35.10	0.91	1	BRC96	UQCRFS1, CCNE1	yes	no
Amp135	19q13.13	43.15-44.48	1.33	1	BRC96	PPP1R14A, SPINT2, MAP4K1, ACTN4, LGALS7, LGALS4, SIRT2	yes	no
Amp136	19q13.2	44.51-44.59	0.08	1	BRC96	PAF1, ZFP36	yes	no
Amp137	19q13.2	44.62-45.05	0.43	1	BRC96	DYRK1B	yes	yes
Amp138	19q13.2	46.65-46.78	0.12	2	BRC104, 148		yes	no
Amp139	19q13.4	59.35-59.37	0.02	1	BRC104		yes	no
Amp140	19q13.42	60.81-60.82	0.01	1	BRC104		yes	no
Amp141	20p13	0.21-0.36	0.15	1	BRC64	TRIB3	no	no
Amp142	20p13	1.91-2.08	0.17	1	BRC64		no	no
Amp143	20p13	2.41-2.59	0.18	1	BRC64		no	no
Amp144	20p12.1	12.98-13.10	0.12	1	BRC64		no	no
Amp145	20p12.1	14.96-17.41	2.46	1	BRC104	PCSK2	yes	yes
Amp146	20q13.13	48.94-49.01	0.07	1	BRC74	ADNP	yes	no
Amp147	20q13.2	49.65-50.20	0.56	1	BRC178	SALL4	yes	no
Amp148	20q13.33	62.04-62.08	0.03	1	BRC82		yes	no
Amp149	21q22.12	33.85-34.48	0.63	1	BRC74	SLC5A3	no	no
Amp150	21q22.3	38.68-39.47	0.80	1	BRC74	ERG	no	yes
Amp151	21q22.3	42.59-42.85	0.26	1	BRC74	TFF3, TFF2, TFF1, TMPRSS3	no	no
Amp152	21q22.3	44.49-45.05	0.56	1	BRC74	DNMT3L	no	no
Amp153	21q22.3	45.18-45.84	0.66	2	BRC74, 104	COL18A1, SLC19A1	no	yes
Amp154	21q22.3	46.37-46.84	0.47	2	BRC74, 104		no	no
Amp155	22q13.1	34.49-34.92	0.43	2	BRC57, 80	APOL3	no	no
Amp156	22q13.2	39.96-40.09	0.12	1	BRC185		no	no
Amp157	22q13.2	41.29-41.30	0.01	1	BRC120		no	yes
Amp158	Xp11.21	55.67-56.33	0.66	1	BRC117	KLF8	yes	yes
HD1	4p14	37.13-37.29	0.16	1	BRC130		yes	no
HD2	4q21.23	85.71-86.67	0.86	1	BRC64	WDFY3	yes	yes
HD3	4q34	175.37-175.65	0.28	1	BRC88		yes	no
HD4	5q31.1	134.26-134.40	0.14	1	BRC104	PITX1	yes	no
HD5	6q27	169.92-169.93	0.01	1	BRC66		yes	no
HD6	7q34	137.92-138.04	0.12	1	BRC130		yes	no
HD7	8p22-p23	10.62-10.74	0.12	3	BRC82, 104, 189	PINX1	yes	no
HD8	8p23.1	11.00-11.14	0.14	1	BRC104		yes	no
HD9	8p22	11.89-15.67	3.78	1	BRC104	DLC1, TUSC3	yes	yes
HD10	8p21	20.88-21.59	0.72	1	BRC104		yes	yes
HD11	8p21	24.27-26.38	2.11	1	BRC104	GNRH1, BNIP3L	yes	yes
HD12	8p21	28.27-29.27	1.00	1	BRC104	FZD3, EXTL3	yes	no
HD13	8p11.21	42.71-43.00	0.29	1	BRC104		yes	no
HD14	9p21	21.31-23.68	2.37	3	BRC88, 119, 170	IFNA2, IFNA1, MTAP, CDKN2A, CDKN2B	yes	yes
HD15	10q23	89.27-89.50	0.23	1	BRC130		yes	no
HD16	10q23.31	90.02-91.03	1.01	1	BRC130	ACTA2	yes	yes
HD17	11p15.4	7.67-7.80	0.13	1	BRC175		no	no
HD18	11q13.5	76.49-76.60.	0.11	1	BRC148		yes	no
HD19	12p13.2	10.98-11.04	0.06	1	BRC117		yes	no
HD20	13q14.3	47.88-49.25	1.37	1	BRC98	RB1, RCBTB2, ARL11, KPNA3	no	no
HD21	15q11.2	20.55-21.24	0.68	1	BRC130	NIPA2	no	yes
HD22	15q11.2	22.77-23.13	0.36	1	BRC130	PAR1, UBE3A	no	no

HD23	15q14	33.60-33.94	0.34	1	BRC117		no	yes
HD24	15q14	35.19-36.41	1.22	1	BRC130		no	yes
HD25	15q21.1	43.45-43.56	0.11	1	BRC117		no	no
HD26	15q15	43.77-45.79	2.02	1	BRC117		no	yes
HD27	15q21.3	53.50-53.62	0.12	1	BRC117		no	no
HD28	16q22.1	67.63-67.71	0.08	1	BRC82		yes	no
HD29	16q22.1	68.75-68.76	0.01	8	BRC62, 64, 106, 110, 120, 160, 164, 189	PDPR	yes	yes
HD30	17p13.2	6.42-6.49	0.06	1	BRC120	TXNL5	yes	no
HD31	18q12.1	26.83-27.38	0.55	1	BRC130	DSC3, DSC2, DSC1, DSG3	yes	yes
HD32	18q21.2	50.11-50.71	0.61	1	BRC82		yes	yes
HD33	22q11.23	22.71-22.72	0.01	5	BRC66, 114, 117, 145, 167	GSTT1	no	yes

Amp: amplification, HD: homozygous deletion.

* Overlap with the Database of Genomic Variants (DGV) entries “Nov 2010” version.

† Overlap with CNV list identified from Koreans [35].

Table S4. Correlation between RARs and clinicopathologic features

		ER Negative	ER Positive	Total	P-value	FDR
RAR-L4	-	21	11	32	<0.001	0.015
	+	2	14	16		
		Stage I	Stage II	Total	P-value	FDR
RAR-G14	-	10	20	30	0.035	0.403
	+	1	17	18		
RAR-G15	-	10	20	30	0.035	0.403
	+	1	17	18		

Table S5. RARs in 48 breast cancers by molecular subtype

RARs	Chr	Cytoband	Total frequency	Luminal A	Luminal B	HER2	TNBC
RAR-G1	1	q21.1	48%	14/29 (48.3%)	4/10 (40.0%)	0/1 (0.0%)	5/8 (62.5%)
RAR-G2	1	q21.2-q21.3	50%	15/29 (51.7%)	4/10 (40.0%)	0/1 (0.0%)	5/8 (62.5%)
RAR-G3	1	q21.3-q23.1	48%	14/29 (48.3%)	4/10 (40.0%)	0/1 (0.0%)	5/8 (62.5%)
RAR-G4	1	q23.2-q23.3	48%	14/29 (48.3%)	4/10 (40.0%)	0/1 (0.0%)	5/8 (62.5%)
RAR-G5	1	q24.13	48%	14/29 (48.3%)	5/10 (50.0%)	0/1 (0.0%)	4/8 (50.0%)
RAR-G6	6	q12	38%	8/29 (27.6%)	6/10 (60.0%)	0/1 (0.0%)	4/8 (50.0%)
RAR-G7	8	q24.13	50%	15/29 (51.7%)	6/10 (60.0%)	0/1 (0.0%)	3/8 (37.5%)
RAR-G8	8	q24.13-q24.21	50%	15/29 (51.7%)	6/10 (60.0%)	0/1 (0.0%)	3/8 (37.5%)
RAR-G9	8	q24.3	50%	13/29 (44.8%)	7/10 (70.0%)	0/1 (0.0%)	4/8 (50.0%)
RAR-G10	8	q24.3	50%	12/29 (41.4%)	7/10 (70.0%)	0/1 (0.0%)	5/8 (62.5%)
RAR-G11	16	p13.3	38%	12/29 (41.4%)	3/10 (30.0%)	0/1 (0.0%)	3/8 (37.5%)
RAR-G12	16	p11.2	40%	10/29 (34.5%)	5/10 (50.0%)	0/1 (0.0%)	3/8 (37.5%)
RAR-G13*	17	q12	38%	7/29 (24.1%)	9/10 (90.0%)	1/1 (100%)	1/8 (12.5%)
RAR-G14	17	q25.1	38%	10/29 (34.5%)	3/10 (30.0%)	0/1 (0.0%)	5/8 (62.5%)
RAR-G15	17	q25.3	38%	11/29 (37.9%)	4/10 (40.0%)	0/1 (0.0%)	3/8 (37.5%)
RAR-G16	20	q13.12	33%	10/29 (34.5%)	2/10 (20.0%)	0/1 (0.0%)	4/8 (50.0%)
RAR-G17	20	q13.33	31%	9/29 (31.0%)	3/10 (30.0%)	0/1 (0.0%)	3/8 (37.5%)
RAR-G18	20	q13.33	31%	8/29 (27.6%)	5/10 (50.0%)	0/1 (0.0%)	2/8 (25.0%)
RAR-L1	8	p23.1-p22	50%	15/29 (51.7%)	5/10 (50.0%)	0/1 (0.0%)	4/8 (50.0%)
RAR-L2	8	p22	48%	14/29 (48.3%)	6/10 (60.0%)	0/1 (0.0%)	4/8 (50.0%)
RAR-L3	8	p21.2	48%	13/29 (44.8%)	6/10 (60.0%)	0/1 (0.0%)	4/8 (50.0%)
RAR-L4	16	q23.1-q23.3	33%	13/29 (44.8%)	1/10 (10.0%)	0/1 (0.0%)	2/8 (25.0%)
RAR-L5	17	p13.1-p12	44%	14/29 (48.3%)	5/10 (50.0%)	0/1 (0.0%)	2/8 (25.0%)

*$P = 1.77 \times 10^{-4}$ based on Fischer's exact test

Table S6. RAR combination rules associated with death events

Rule for death event	Laplace accuracy
RAR-G1(+), RAR-G7(+), RAR-G12(+), RAR-G13(+)	0.83
RAR-G5(-), RAR-G7(+), RAR-G12(+), RAR-G13(+)	0.80
RAR-G5(-), RAR-G8(+), RAR-G12(+), RAR-G13(+)	0.80
RAR-G5(-), RAR-G9(+), RAR-G12(+), RAR-G13(+)	0.80
RAR-G5(-), RAR-G10(+), RAR-G12(+), RAR-G13(+)	0.80
RAR-G5(-), RAR-G11(+), RAR-G12(+), RAR-G13(+)	0.75
RAR-G5(-), RAR-G17(+), RAR-G12(+), RAR-G13(+)	0.75
RAR-G5(-), RAR-L5(+), RAR-G12(+), RAR-G13(+)	0.75

Table S7. Frequency of chromosomal arm changes in 48 breast cancers.

Chr arm	Gains	Losses		Chr arm	Gains	Losses
1p	8	0		13p	0	0
1q	23 (47.9%)	0		13q	0	0
2p	0	6		14p	0	0
2q	0	9		14q	6	15 (31.3%)
3p	0	11		15p	0	0
3q	9	0		15q	7	12
4p	6	9		16p	18 (37.5%)	0
4q	0	12		16q	8	16 (33.3%)
5p	10	0		17p	0	21 (43.8%)
5q	6	14		17q	18 (37.5%)	0
6p	11	0		18p	0	0
6q	18 (37.5%)	13		18q	0	0
7p	7	0		19p	0	3
7q	9	7		19q	13	5
8p	7	23 (47.9%)		20p	0	0
8q	24 (50.0%)	0		20q	16	0
9p	0	10		21p	0	0
9q	8	0		21q	10	0
10p	0	0		22p	0	0
10q	0	11		22q	10	0
11p	0	12		23p	0	14
11q	9	14		23q	5	14
12p	10	11		24p	0	0
12q	8	0		24q	0	0

Note: Gray boxes represent frequent chromosomal arm changes (> 30%)
